# Oral contraceptives cause evolutionarily novel increases in hormone exposure: A risk factor for breast cancer

**DOI:** 10.1093/emph/eox009

**Published:** 2017-06-05

**Authors:** Jennie L Lovett, Margo A Chima, Juliana K Wexler, Kendall J Arslanian, Andrea B Friedman, Chantal B Yousif, Beverly I Strassmann

**Affiliations:** 1 Department of Anthropology, University of Michigan, Ann Arbor, MI 48109, USA; 2 Research Center for Group Dynamics, Institute for Social Research, University of Michigan, Ann Arbor, MI 48106, USA

**Keywords:** estrogen, progesterone, oral contraceptives, breast cancer

## Abstract

**Background and objectives:**

In the evolutionary past, women spent most of their reproductive lives either pregnant or in lactational amenorrhea, and rarely menstruated. The current pattern of frequent menses, and the associated increase in endogenous hormonal exposure, has been implicated in the current breast cancer epidemic. It is not known, however, whether oral contraceptives further increase, or actually decrease, hormonal exposure over one menstrual cycle. Here, we examined variation in hormonal exposure across seven oral contraceptive (OC) formulations, and produced the first quantitative comparison of exogenous versus endogenous hormone exposure.

**Methodology:**

Data from 12 studies of serum estradiol (E2) and progesterone (P4) were aggregated to create a composite graph of endogenous hormone levels over one menstrual cycle in European or American women (age 19–40 years). Pharmacokinetic package insert data, also from Western women, were used to calculate exposures for hormones in seven different OC formulations. Endogenous and exogenous hormone levels were compared after adjusting for the relative binding affinity (RBA) of progestin to the progesterone receptor and ethinyl estradiol (EE) to the estrogen receptor.

**Results:**

After adjusting for RBA, median ethinyl estradiol exposure across 28 days in the OCs was 11.4 nmol/l, similar to median E2 exposure. One formulation, however, was 40% higher in ethinyl estradiol exposure relative to median endogenous estradiol. Median exposure from progestins in OCs (1496 nmol/l) was 4-fold higher than the median endogenous exposure from P4 (364 nmol/l). Exposure from OC progestins ranged from one sixtieth to 8-fold median endogenous P4 over 28 days.

**Conclusions and implications:**

Given that breast cancer risk increases with hormonal exposure, our finding that four widely prescribed formulations more than quadruple progestin exposure relative to endogenous progesterone exposure is cause for concern. As not all formulations produce the same exposures, these findings are pertinent to contraceptive choice. We also identify critical gaps in the provision of relevant data on pharmacokinetics and carcinogenicity by drug manufacturers.

## Teaser

Several oral contraceptive pills cause evolutionarily novel increases in hormone exposure and may thereby promote breast cancer risk. Half of all formulations in this study increase hormone levels more than four times what a woman would get from her own ovaries if not on the pill.

## INTRODUCTION

Breast cancer is the most frequent malignancy and the second leading cause of cancer death in American women [1]. Comparison of the life history traits of women in natural fertility populations [2] with those of women in modern societies has helped to understand the origins of the breast cancer epidemic [3, 4]. A useful comparison population for American women is the Dogon of Mali, whose reproductive biology has been studied for 30 years [2, 5, 6] and whose life history traits have been extensively characterized [7].

Among the Dogon, the median age at menarche is 17 years versus 12 years for girls in the United States [8], a difference of five years in exposure to ovarian hormones. Epidemiological evidence demonstrates a correlation between levels of endogenous sex hormones and risk for breast cancer [9–11]. The median age at first full term pregnancy among Dogon women is 19 years versus 26 years for women in the United States [12]–a seven year delay in the maturation of the breast lobules, which undergo permanent protective changes in cellular and molecular composition upon maturation [13]. The median duration of lactational amenorrhea in Dogon women is 20 months [5] compared to 6 months for a study of breastfeeding women in the United States [14]. Lactational amenorrhea is a time of ovarian quiescence when the steroid hormones estrogen and progesterone remain at baseline compared with the peaks and troughs associated with menstrual cycling [15]. Dogon women experience about 100 menses per lifetime, which is 4-fold lower than for women in the United States [2], a significant difference given that breast cancer risk increases with the number of menses [16]. Dogon women have a median of nine live births [7, 17], versus two for parous women in the United States [12]. Nulliparity is rare in Dogon women (<1%), whereas in 2014, 17% of American women in the cohort aged 45 and 50 years had not reproduced [18].

Associations between parity and breast cancer risk are complex and depend on age, race and breastfeeding behavior; however, it is well established that nulliparous women and women who first give birth at later ages are at higher risk for breast cancer [19–22].

The life history traits of women in natural fertility populations like the Dogon of Mali are probably characteristic of women during the evolutionary past before the demographic transition to low fertility [2, 3, 23, 24]. In natural fertility populations, endogenous exposure to ovarian steroid hormones is lower than in modern populations where fertility is limited to one or two livebirths [24]. Two possible mechanisms provide support for a dose-dependent relationship between ovarian steroid hormones and breast cancer risk. First, the natural estrogens (estrone and estradiol) are reported to be mutagenic and carcinogenic through a genotoxic mechanism–formation of depurinating estrogen-DNA adducts by the reaction of catechol estrogen quinones with DNA [25]. Second, the mechanism may also involve the stimulatory effect of estrogen and progesterone on cell proliferation in the breast, potentially via breast tumor stem cells [26, 27]. Regardless of which mechanism is more important, there is good cause to examine any lifestyle factor that increases hormonal exposure.

Here we ask: Has the change in hormonal exposure and breast cancer risk due to modern life history patterns been further aggravated by the use of hormonal contraceptives? The most common type of hormonal contraceptive combines two synthetic compounds, ethinyl estradiol and a progestin, to prevent ovulation by suppressing the lutenizing hormone (LH) surge [28]. These ‘combination oral contraceptives’ (OCs) are taken by more than 100 million women worldwide [29] including 9.7 million women in the United States [30]. Despite the widespread use of OCs, no previous study has compared exogenous exposure from OCs to the endogenous exposure to estradiol (E2) and progesterone (P4) experienced by regularly cycling women. We compared the variation in hormonal exposure across seven different OC regimens, and provide the first quantitative comparisons of this exogenous exposure to the endogenous hormonal exposure from ovulatory menstrual cycles in women age 19–40 years over a 28 day menstrual cycle.

One way to evaluate breast cancer risk from OCs is to measure the relative risk (RR), often approximated as the odds ratio (OR), for breast cancer in women who had ever used OCs versus never-users. A meta-analysis of 13 prospective cohort studies found a nonsignificant positive association between OC use and breast cancer risk (RR 1.08, 95% Cl 0.99–1.17) [31]. In the same study, a dose-response analysis based on five studies showed that the risk of breast cancer increased significiantly by 7% (RR 1.07, 95% Cl 1.03–1.11) and 14% (RR 1.14, 95% Cl 1.05–1.23) for every five and ten year increment of OC use, respectively. Another meta-analysis of case-control studies found that OC use was associated with 29% higher breast cancer risk in parous women (OR 1.29, 95% CI, 1.20–1.40), 24% higher risk in nulliparous women (OR 1.24, 95% CI 0.92–1.67) and 19% higher risk in women younger than 50 years (OR 1.19, 95% CI 1.09–1.29) [31]. In parous women who used OCs before their first full-term pregnancy, risk for breast cancer increased by 44% (OR 1.44, 95% CI 1.28–1.62), and by 52% if OC use lasted for four or more years (OR 1.52, 95% CI 1.26–1.82) [31]. A recent case-control study reported that current OC users (≥ 5 years and ages 20–39 years) had an increased risk for estrogen-receptor negative (ER^-^) and triple-negative breast cancer (ER^**−**^: OR 3.5, 95% CI 1.9–9.0; triple-negative: OR 3.7, 95% CI 1.2–11.8) [32]. Studies of the effect of duration of OC use on breast cancer risk were more likely to find a strong, positive association in younger women [32, 33]. Given the tendency for women to start using OCs at younger ages and to use them for longer intervals prior to first birth, the results of the above studies are cause for concern [31].

Another major analysis is the Women’s CARE study [34], which found no evidence that specific OC formulations increase breast cancer risk in women 35–64 years of age. However, lumping women together whose ages ranged from 35 to 64 years might have obscured risks accruing only to the youngest age cohorts; moreover women under age 35 years were not part of the study. The breast cancers were diagnosed in the period 1994–98 and this study was similar to another [21] in that it did not include the newer contraceptives. Further, it was subject to the limitations of small sample sizes for OCs of a given formulation.

Data are presently not available that would enable a comparison of the breast cancer risks associated with the different OC formulations in wide use today, and larger studies are needed to replicate preliminary findings that breast cancer risk differs by OC formulation [33, 35, 36]. Inevitably, epidemiological research on OCs and breast cancer risk will suffer from time-lags and will not reflect exposures from the newer contraceptives in use at any given time point. It is therefore helpful to consider theoretical risks associated with increases in hormonal exposure associated with particular OC formulations. The life history patterns of modern women already have increased hormonal exposure well above that of women in natural fertility populations–making it important to know whether hormonal contraceptives further aggravate, or attenuate, the risks.

## METHODOLOGY

### Endogenous hormone exposure

To determine endogenous hormone levels we searched PubMed and Web of Science for published studies of serum levels of estradiol (E2) and progesterone (P4) in regularly cycling women. We included only those studies in which the cycle data were aligned with respect to the luteinizing hormone (LH) peak, defined as cycle day 0. This search identified 12 studies of 181 women in the United States and Europe whose 302 ovulatory cycles were used in the analysis [37–48]. Characteristics of the women, sampling frequencies, and other study details are summarized in Supplementary Table S1. The precise definition of ‘regular cycling’ was variable, but meant that the women were experiencing normal menstrual cycles with ovulation and menstruation occurring on a monthly basis. Evidence of ovulation ranged from luteinizing hormone peaks to visualization of follicles using ultrasound. We excluded women younger than 19 years and older than 40 years in order to reduce the most extreme age-related hormone changes.

We used the published graphs in the 12 studies to obtain E2 and P4 serum levels for each day of the menstrual cycle and we aggregated the data using Microsoft Excel to create a composite graph of E2 and P4 concentrations over 28 days, aligned with respect to the LH peak. We converted all units to pmol/l of serum using the molecular weight of 272.38 g/mol for E2 and 314.46 g/mol for P4 [49]. We then estimated the total hormone exposure for both E2 and P4 by measuring the area under the curve using the linear trapezoidal method [50].

### Exogenous hormone exposure

To identify a set of widely prescribed hormonal contraceptives at a well-defined location serving young women, we contacted the University Health Service (UHS) at the University of Michigan. The UHS provided us with a list of the 10 most frequently dispensed hormonal contraceptives at the UHS pharmacy (Table 1). We excluded four OCs from our analysis due to missing information in the package inserts and elsewhere for maximum serum concentration (C_max_) for days other than the first day of dosing. We also excluded a vaginally inserted hormone-releasing ring that lacked a C_max_ value for the first day of dosing and that was not directly comparable to the orally administered OCs. We included formulas for Yaz (Bayer HealthCare Pharmaceuticals, Inc., Table 1, G) and Ortho Tri-Cyclen Lo (Ortho-McNeil-Janssen Pharmaceuticals, Table 1, E) in our analysis, although they were not on the UHS list in 2011, based on previous widespread use in the United States. The resulting list included seven OCs, all of which contained EE and a progestin in a variety of different dosing regimens (Table 1). The necessary parameters for our analysis of each hormone in each OC were serum C_max_ values for at least the first and last days of dosing, excretion half-life values, molar mass, and exposure for the first 24 hr given by the area under the curve for the first day (AUC_0__–__24 h_).

**Table 1.** List of widely dispensed hormonal contraceptives

Formulation	Progestin	Progestin mg/day (days dosage)	Ethinyl Estradiol mg/day (days dosage)	Generation^c^
**A**	levonorgestrel	0.100 (21)	0.020 (21)	2^nd^
**B**	norethindrone acetate	1.000 (24)	0.020 (24)	2^nd^
**C**	desogestrel	0.150 (21)	0.020 (21)	3^rd^
			0.010 (5)	
**D**	norgestimate	0.250 (21)	0.035 (21)	2^nd^
**E**^a^	norgestimate	0.180 (7)	0.025 (21)	2^nd^
		0.215 (7)		
		0.250 (7)		
**F**	drospirenone	3.000 (21)	0.030 (21)	4^th^
**G**^a^	drospirenone	3.000 (24)	0.020 (24)	4^th^
**–**^b^	norethindrone acetate	1.5 (21)	0.030 (21)	2^nd^
**–**^b^	norethindrone acetate	0.350 (continuous)	–	2^nd^
**–**^b^	norgestimate	0.180 (7)	0.035 (21)	2^nd^
		0.215 (7)		
		0.250 (7)		
**–**^b^	etonogestrel vaginal ring	0.120 (continuous)	0.015 (continuous)	
**–**^b^	desogestrel	0.150 (21)	0.030 (21)	3^rd^

a Not included in UHS list of commonly prescribed contraceptives but included in analysis.

b Not included in analysis due to lack of pharmacokinetic information in the package inserts.

c Generation identified based on criteria in Golobof and Kiley [28].

Package inserts for only two of the OCs [Ortho Tri-Cyclen Lo (Ortho-McNeil-Janssen Pharmaceuticals, Table 1, E) and Alesse (Wyeth Pharmaceuticals, Inc., Table 1, A)] included both EE and progestin C_max_ concentrations for an additional day rather than only the first and last days of dosing. We established that for a linear regression of C_max_ against time in days, the logarithm of C_max_ yielded the highest R squared value for both Ortho Tri-Cyclen Lo and Alesse. [The R squared values for Ortho Tri-Cyclen Lo were 0.98 (EE) and 0.97 (progestin) and for Alesse were 0.97 (EE) and 0.99 (progestin).] We therefore used logarithmic regression to fit the pharmacokinetic data for the other five OC regimens for which the package inserts (Loestrin 24 FE, Actavis Inc. = B; Mircette, Teva Pharmaceuticals, Inc. = C; MonoNessa, Actavis Inc. = D, Yasmin, Bayer Corp. = F; Yaz, Bayer Corp. = G) reported serum levels for only the first and last days of dosing. From the equation for the best-fit line, we calculated the maximum serum level for each day of the dosing period (usually 21 or 24 days).

We converted serum hormone concentrations from pg/ml to pmol/l by dividing the serum concentration for a given hormone by its molar mass and multiplying by 1000 [49]. We used the linear trapezoidal rule for numerical integration to calculate the exposure for each hormone during the dosing period [50]. Hormonal exposure from ingestion of the first pill to C_max_ on the first day of dosing was calculated by converting the area under the curve for the first day (AUC_0-24 h_), given on the package inserts in either pg/ml or ng/ml, into pmol*day/l. We calculated daily hormone exposure from the last day of dosing (usually either cycle day 21 or 24) to day 1 of the next 28 day cycle by using the half-life excretion values provided by the pharmaceutical companies and by assuming exponential decay [51]. Total hormone exposure during these non-dosage days was calculated using the linear trapezoidal method to determine the area under the curve [50]. Total exposure over 28 days in nmol/l was based on the sum of: (1) exposure from ingestion of the first pill to C_max_ for the first day of dosing, (2) exposure from the first to last day of dosing and (3) exposure after the last day of dosing.

Relative binding affinity (RBA) is used to estimate the binding affinity of a ligand for a receptor and is a measure of the concentration of ligand that competes for half the total specific binding [52]. We compiled 15 *in vitro* studies that report the RBAs of progestins to the progesterone receptor (PR) and, to our knowledge, these are the only studies in the primary literature that compare the RBAs for the progestins in our list of OCs (Supplementary Table S2). We excluded studies that did not use human uterine tissue as the source of the progesterone receptors and we also assembled major review articles that report RBAs for progestins (Supplementary Table S3). We standardized the progestin exposure from the various OCs by multiplying each progestin exposure (nmol/l) by the RBA of its progestin to the progesterone receptor divided by 100, which is the RBA of progesterone to the progesterone receptor (details in Table 2). Similarly, we multiplied the EE exposure (nmol/l) by the RBA of EE to the estrogen receptor (RBA = 190), divided by 100. Thus, to compare hormone exposure from the EE in OC formulations to that derived from E2 over 28 days, we multiplied EE exposure for each OC by a factor of 1.9.

**Table 2.** Total exogenous exposure over 28 days from ethinyl estradiol (column 2) and progestin in OCs (column 5) compared to total endogenous exposure to estradiol and progesterone in a 28 day menstrual cycle (bottom two rows)

1	2	3	4	5	6	7
Combination oral contraceptive^a^	Unstandardized ethinyl estradiol exposure (nmol/l)	Relative Binding Affinity of ethinyl estradiol to the estrogen receptor^b^	Ethinyl estradiol exposure standardized by RBA (nmol/l)	Unstandardized progestin exposure (nmol/l)	Relative Binding Affinities (RBAs) of the progestins to the progesterone receptor^c^	Progestin exposure standardized by the median RBA (nmol/l)
A	5.7	190	10.7	368	300, 323, 628, 671, 833, 1500	2390
B	6.3	190	12.0	997	98, 134, 150, 193, 306	1496
C	4.8	190	9.1	248	2, 2.5, 3.3	6
D	9.5	190	18.1	294	2.3, 30	47
E	6.4	190	12.2	244	2.3, 30	39
F	6.0	190	11.4	4479	19, 67, 80	3001
G	3.1	190	5.7	3735	19, 67, 80	2502
Mean (SD)	6.0 (1.93)		11.3 (3.74)	1480.71 (1825.79)		1354.46 (1315.17)
Median	6.0		11.4	368		1496
**Endogenous menstrual cycle**	**Estradiol exposure (nmol/l)**			**Progesterone exposure (nmol/l)**		
Mean (SD)	12.76 (1.81)			393.99 (67.90)		
Median	12.97			363.75		

To produce the standardized ethinyl estradiol exposure (column 4), we adjusted for the difference in RBA between natural estradiol and synthetic ethinyl estradiol for the estrogen receptor (which have RBAs of 100 and 190, respectively). To produce the standardized progestin exposure (column 7), we adjusted for the difference in RBA of natural progesterone to the progesterone receptor, which is 100, and the RBAs of the various progestins to the progesterone receptor, which vary widely (column 6). In this calculation, we used the median of the reported RBA values for a given progestin to the progesterone receptor.

a Defined in Table 1.

b RBA values from [54]. Sprague-Dawley rats are the source of estrogen receptor.

c RBA values from Supplementary Tables 2 and 3. RBAs are from the following sources: 300 = (Pollow *et al.* 1989); 323 = (Philibert *et al.* 1999); 628 = (Bergink *et al.* 1981); 833 = (Pollow *et al.* 1992; Juchem *et al.* 1993); 1500 = (Bergink *et al.* 1981); 98 = (Kasid *et al.* 1978); 134 = (Philibert *et al.* 1999); 150 = (Kuhl 2001) (original source unknown); 306 = (Bergink *et al.* 1981); 2 = (Kuhl 2001) (original source unknown); 2.5 = (Pollow *et al.* 1989); 3.3 = (Juchem *et al.* 1993); 2.3 = (Juchem *et al.* 1993); 30 = (Kuhl 2001) (original source unknown); 19 = (Krattenmacher 2000); 67 = (Pollow *et al.* 1992); 80 = (Kuhl 2001) (original source unknown).

## RESULTS

**Figure 1.**
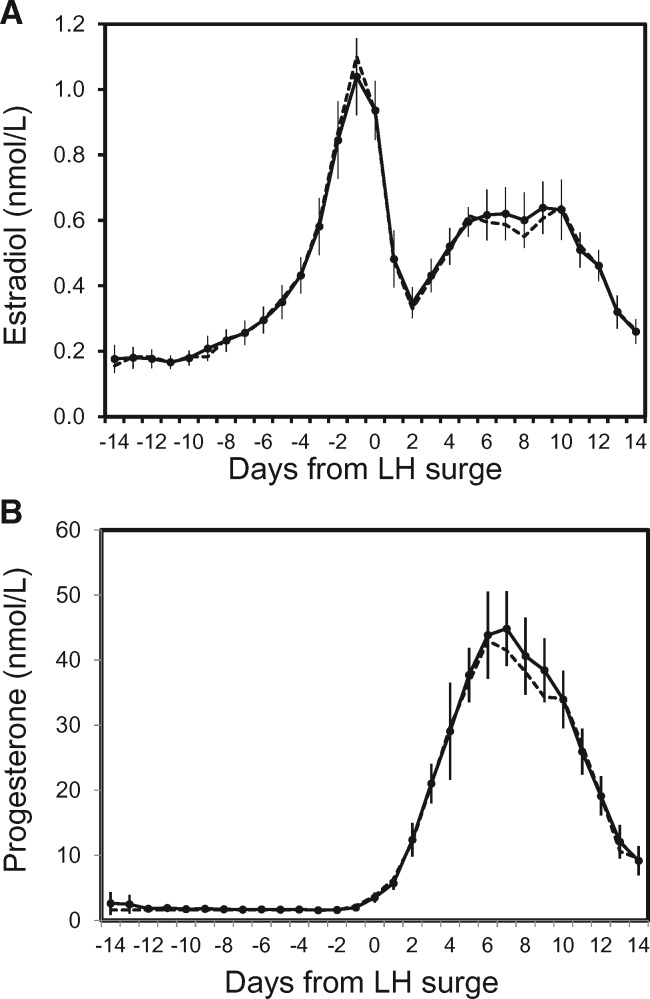
(**A**) Daily endogenous estradiol exposure observed over 28 days in nmol/l based on data from 12 studies of women in the US and Europe (*N* = 181 women and 302 ovulatory cycles). Solid line and circles are the mean endogenous estradiol exposure with 95% confidence intervals. Dashed line is median exposure. (**B**) Daily endogenous progesterone exposure observed over 28 days in nmol/l based on data from 12 studies. Solid line and circles are the mean endogenous progesterone exposure with 95% confidence intervals. Dashed line is median exposure

A total of 181 American and European women contributed 302 menstrual cycles to the analysis of endogenous estradiol and progesterone levels. The composite graph of E2 and P4 concentrations over a 28 day menstrual cycle displayed the classic shape and hormone concentrations for ovulatory cycles, with an E2 peak the day prior to the LH surge, and a lower, but wider peak in the luteal phase (Fig. 1). P4 peaked 7 days after the LH surge. On a given cycle day, mean and median hormone concentrations were very similar (Fig. 1).

Prior to standardizing by RBA, the total exposure to EE over 28 days was lower in all seven OCs than was exposure to endogenous E2 over the same time period (Table 2, Fig. 4A). The median total exposure to EE in the OCs was 6.0 nmol/l and the median total endogenous exposure to E2 was 12.97 nmol/l, which is more than double that of the OCs (Table 2: column 2). Variation in unstandardized EE exposure was 3-fold from the OC with the lowest to the highest exposure (Fig. 4A). After adjusting for the difference in the binding affinity of E2 and EE to the estrogen receptor (Table 2: column 4), the median exposure from EE in the OCs was 11.4 nmol/l, which was similar to E2 exposure over one menstrual cycle.

Compared to the median endogenous P4 exposure, the OC containing levonorgestrel (A) produced an exposure similar to endogenous P4 prior to standardizing for differences in RBA. Total progestin exposure was lower in the three OCs that contained desogestrel (C) or norgestimate (D, E), and was higher in the three OCs that contained norethindrone (B) or drospirenone (F, G) (Table 2: column 5, Fig. 3, Fig. 4B). Exposure from the formulation with the highest progestin dose [drospirenone 3.0 mg/day with EE 0.03 mg/day (F)] was 18 times higher than for the two OCs with the lowest exposure [desogestrel (C) and norgestimate (E)] and 12 times higher than the median endogenous exposure to P4 (363.75 nmol/l) (Table 2: column 5).

After standardizing for differences in RBA, the median exposure from progestin in the OCs (1496 nmol/l) was 4-fold higher than the median endogenous exposure from P4 over 28 days (363.75 nmol/l). The range in standardized exposures for the OCs over 28 days was very wide: exposure from desogestrel (C) was only one sixtieth of median endogenous exposure from P4, whereas the two formulations with drospirenone (G, F) gave exposures that were 7- and 8-fold higher than median endogenous P4.

**Figure 2.**
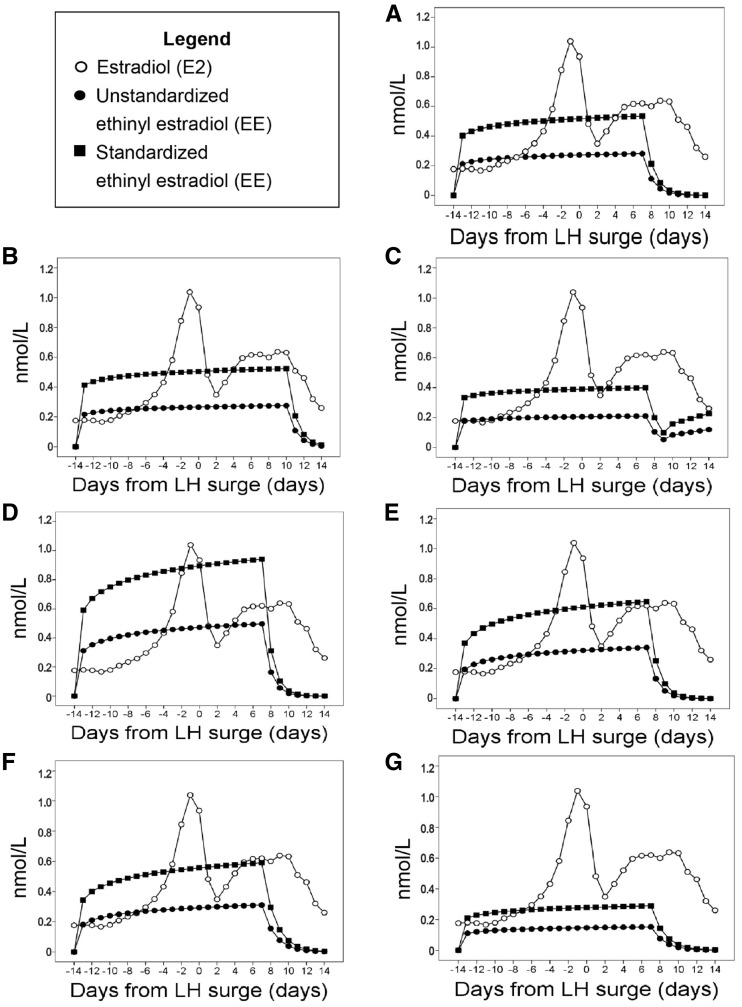
Daily 17-β-estradiol exposure in non OC-users compared to daily ethinyl estradiol exposure, observed over 28 days measured in nmol/l. Open circles are endogenous E2 daily exposure, solid circles are unstandardized exogenous EE daily exposure and solid squares are exogenous EE exposure standardized by the RBA of EE to the estrogen receptor, which is 190. EE exposure is reported for each of seven OC formulations detailed in Table 1

**Figure 3.**
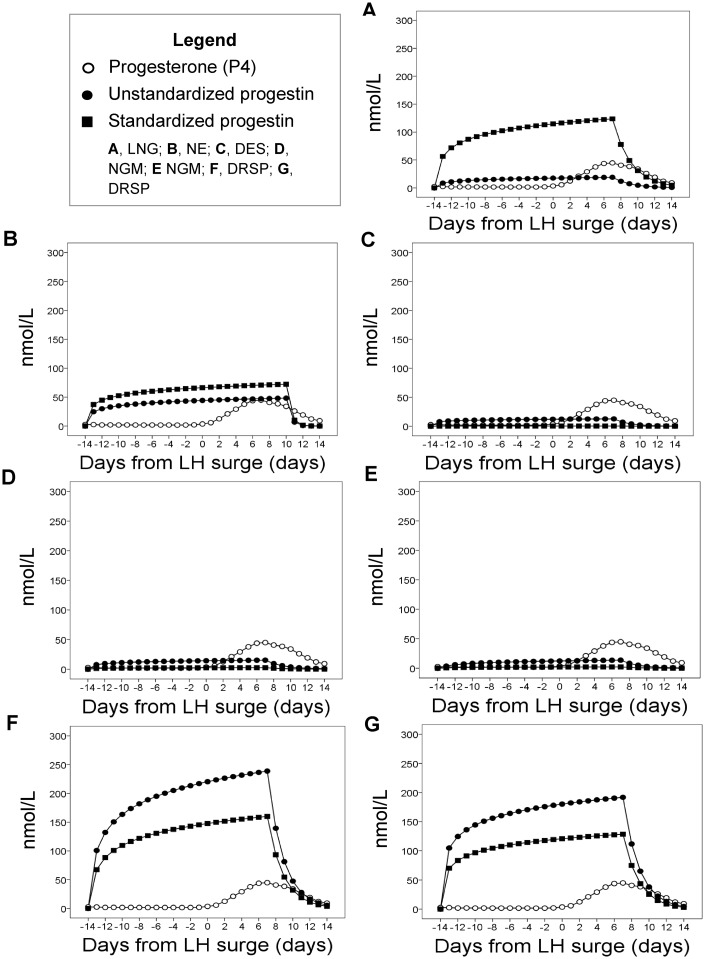
Daily progesterone exposure in non OC-users compared to daily progestin exposure, observed over 28 days, measured in nmol/l. Open circles are endogenous P4 daily exposure, solid circles are unstandardized exogenous progestin daily exposure and solid squares are exogenous progestin exposure standardized by the RBA of each progestin to the progesterone receptor, which varies for each progestin (Table 2). Progestin exposure is reported for each of seven OC formulations detailed in Table 1. There are five types of progestins: levonorgestrel (LNG), norethindrone (NE), desogestrel (DES), norgestimate (NGM) and drospirenone (DRSP)

The change in amplitude for hormone exposure over 28 days was much greater for endogenous E2 than for EE in all seven OC regimens; EE exposures for the OCs were quite flat from cycle day –13 to +7 (Fig. 2). Similarly, in the OCs with relatively low total progestin exposure, progestin values were constant across most cycle days and did not generate the characteristic progesterone peak seen during the luteal phase of ovulatory menstrual cycles (Fig. 3). By contrast, the change in amplitude for progestin exposure was quite dramatic in the two OCs that contained drospirenone (F, G) and their peak was far higher than the endogenous luteal peak in P4 (Fig. 3).

**Figure 4.**
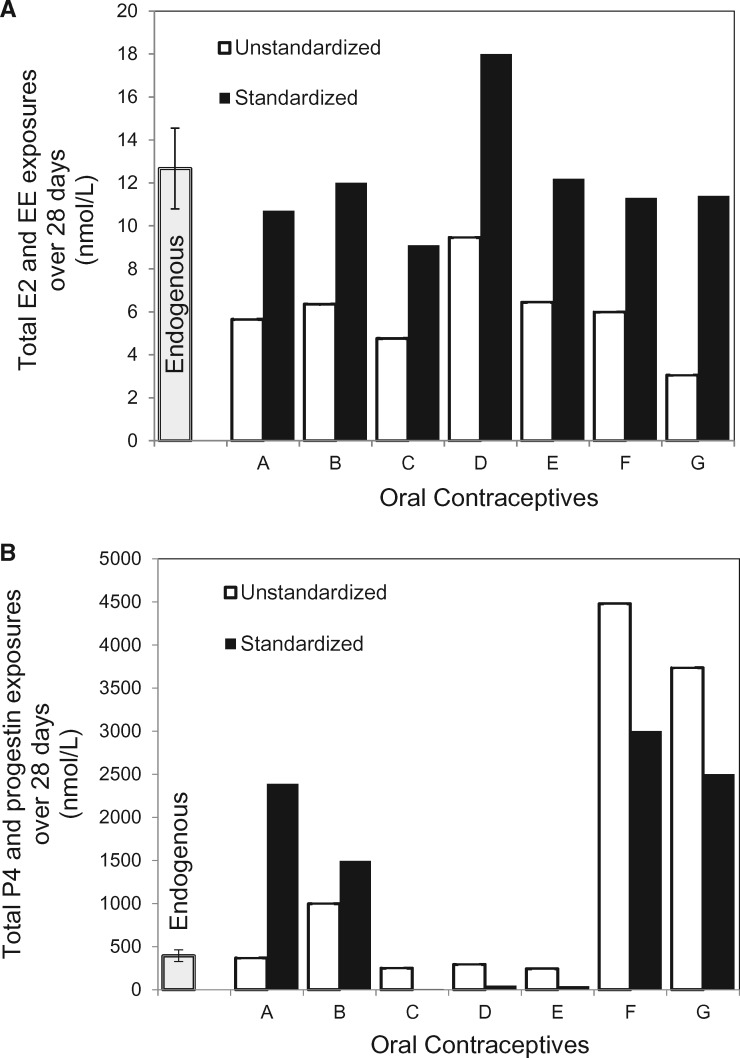
(**A**) Over a 28-day cycle, total mean ± SD in nmol/l of endogenous 17-β-estradiol exposure in non-OC-users compared to total exogenous EE exposure from women taking each each of seven OC formulations detailed in Table 1. Open bars are unstandardized EE exposure, and solid bars are EE exposure standardized by the RBA of EE to the estrogen receptor. (**B**) Over a 28-day cycle, total mean ± SD in nmol/l of endogenous progesterone exposure in non-OC-users compared to exogenous progestin exposure in women taking each OC (A-G). Open bars are unstandardized progestin exposure, and solid bars are progestin exposure standardized by RBA of each progestin to the progesterone receptor (Table 2)

## DISCUSSION

Our study compared hormonal exposure from seven different OC regimens to endogenous hormonal exposure from ovulatory menstrual cycles in women aged 19–40 years.

After correcting for the difference between E2 and EE in RBA to the estrogen receptor, the median exogenous exposure was 11.4 nmol/l, similar to the median endogenous exposure of 12.97 nmol/l (Table 2). Among different OCs, exposures ranged from 5.7 nmol/l for the OC with the least EE to 18.1 nmol/l for the OC with the most EE (Table 2: column 4). Although there was one exception (Formulation D or Sprintec), the OCs we examined do not increase ethinyl estradiol exposure beyond what a woman would experience from endogenous E2 if she were not taking OCs. However, future research is needed that directly compares the carcinogenicity of EE and E2 in humans.

After standardizing for differences in RBA, the progestin exposure from several formulations was higher than the median endogenous P4 exposure of 363.75 nmol/l. Specifically, the formulation containing norethindrone (B) gave 4-fold higher exposure, the formulation containing levonorgestrel (A) gave over 6 times more exposure and the two formulations containing drospirenone gave exposures that were approximately 7-fold (G) and 8-fold (F) higher than the median endogenous P4 exposure. On the other hand, exposure from desogestrel (C) was only one sixtieth of median endogenous exposure from P4.

In sum, OCs that contain levonorgestrel, norethindrone, or drospirenone resulted in progestin exposure that is high in relation to endogenous P4. This result provides a theoretical basis for predicting that these OCs are an environmental risk factor for breast cancer. At a minimum, they suggest the urgent need for further evaluation of absolute and relative hormonal exposures from various OCs. The shape of the concentrations over a cycle differs for endogenous and exogenous hormones, which could influence whether premalignant cells undergo apoptosis upon hormone withdrawal. Thus, further research is needed on the safety of the various formulations now used by millions of women. The various formulations we studied produce strikingly different exposures, leading us to predict that they also differ from each other in their cancer risks.

The estrogen receptor is expressed in 75% of breast cancers and plays a major role in breast cancer development and progression [53]. An important strategy in endocrine therapies for breast cancer is the use of antagonists to prevent estrogen from binding to the estrogen receptor [53]. The binding affinity of synthetic EE to the estrogen receptor is 90% higher than the binding affinity of natural E2 [54], and EE is a far more potent hormone [55]. EE increases breast epithelial cell proliferation in a manner that is dose-dependent and increases risk for breast cancer in women [55, 56]. Although the carcinogenic effects of EE on the breast are not entirely mediated by the estrogen receptor as other receptors and pathways are also involved, the role of the estrogen receptor is pivotal [53].

The progesterone receptor is expressed in 50–70% of primary tumors, and there is evidence that progesterone exposure plays a role in breast carcinogenesis even though the precise mechanisms remain undetermined [57, 58]. Progesterone antagonists (antiprogestins) and progesterone receptor modulators (PRMs) are used in the treatment of PR^+^ breast cancer [59], and antagonism at the PR helps protect against breast cancer. Adjusting for the RBA of the various progestins to the progesterone receptor is necessary for gauging their biological activity. OCs containing norethindrone or drospirenone gave much higher exposures than P4 regardless of whether we standardized for RBA or not. However, exposure from the formulation containing levonorgestrel, which is an extremely potent hormone, was not particularly high prior to correcting for RBA. This result highlights the need to adjust for RBA in order to compare different compounds. One cannot consider merely the amount of hormone analog in the various formulations. One must also consider differences in potency, and this information is not provided in the package inserts accompanying OC prescriptions nor is it readily available to health care providers.

Nonetheless, there are several limitations to using RBA as a measure of potency. The first is that RBA is only an approximation of the biological activity of the hormone-receptor complex. Assessing binding is further complicated by progesterone receptor isoforms (either PR-A or PR-B) that occur in different ratios in different reproductive tissues [52]. The second limitation is that all RBAs were measured using receptors from uteri and the biological activity of the hormone-receptor complex is different in breast and endometrial tissue. Additionally, progestin potencies may be underestimated because RBAs are measured in the absence of estrogen [60]. In studies that measured the number of days menses were delayed, the addition of estrogen to a progestin increased the potency of the progestin markedly [60]. Furthermore, each progestin has a unique RBA not only to the PR, but also to several other steroid receptors (androgen, estrogen, glucocorticoid and mineralocorticoid receptors), which we did not include in our analysis (Supplementary Table S4). The bioavailability and the ovulation inhibitory dose also vary among progestins (Supplementary Table S4).

Another area of complexity is the natural variation that occurs in endogenous ovarian steroid hormones. Natural variation in sex steroid hormone levels occurs among populations, among women from the same population, among different menstrual cycles for a given woman and within menstrual cycles [61–63]. The factors that influence this variability are numerous and include genes, early developmental conditions and adult lifestyle [61]. Among the lifestyle influences, energetic factors are especially important for ovarian function. Reductions in caloric intake, weight loss and increased energy expenditure were associated with reduced ovarian steroid hormone production within populations in several countries such as Nepal [64], Congo [65] and Poland [61, 66, 67]. Comparisons of interpopulation differences in ovarian function have suggested that women in the United States may tend to have relatively high ovarian function [61, 68, 69] although in some cases the differences may be at least partially influenced by methodological issues such as different sampling protocols beween studies [62].

Here, we restricted our comparison of exogenous and endogenous hormone exposure to data sets for Western women from the United States and Europe (Supplementary Table S1), which limits the generalizability of our results. We did not attempt to compare the exogenous exposure in Western women from the package inserts to the global range of natural variation in hormone levels. Such a goal is beyond our scope, especially since data for women in mid and low income countries was usually from specimens of saliva, urine, or less commonly, blood spots [62]. To be consistent, we compared exogenous and endogenous values in serum. The Western women in our study were ‘healthy’ and presumably not under energetic stress (See Supplementary Table S1), making it quite possible that they tended to have higher endogenous hormone profiles than women in energetically stressed populations. If that is the case, then our analysis is conservative in regard to the conclusion that several of the OCs we examined increase hormone exposure. Women in under-nourished populations may incur an even greater increase from endogenous to exogenous hormone exposure when they take OCs, which might exacerbate their breast cancer risk. It has already been reported that when endogenous hormone exposure is low, women who take OCs may experience more side effects [70, 71]. In sum, our study does not identify the individual risk level that a woman may experience upon taking OCs, but its findings should be applicable to a broad range of women. Moreover, it is not part of standard practice for endogenous hormone levels to be assayed when OCs are prescribed [72].

## CONCLUSIONS AND IMPLICATIONS

More robust methods are needed for comparing the cancer risks from endogenous and exogenous hormonal exposures. Exogenous exposures could be more accurately calculated if OC manufacturers would provide serum hormone concentrations on each of the 28 days of the menstrual cycle, rather than the current practice of providing only two or three C_max_ values for the entire cycle. These daily serum hormone concentrations would improve calculations of total exposure over the course of the month and better depict the shape of the exposure curve. In package inserts, OC manufacturers report data from sample sizes of 12–79 individuals, which is unlikely to be a large enough sample to capture the substantial within and between population heterogeneity in the bioavailability of the various progestins and ethinyl estradiol (Supplementary Table S4) [73]. The OC manufacturers should also provide more extensive documentation on the potency of each hormone in their formulations. Case-control and cohort studies need to evaluate breast cancer risk from the most recent OC formulations, with particular focus on the comparative risks associated with different OCs. In the meantime, our results accentuate concerns about the safety of several commonly prescribed hormonal contraceptives–especially for young nulliparous women.

From a comparison of prescribing practices for OCs at UHS in 2010 and 2016, we infer a high degree of stability in the prescriptions for hormonal contraceptives at this health facility over time. The top three most prescribed OCs were the same in both 2010 and 2016 (Junel FE 1/20, Tri-Sprintec and Sprintec). Junel FE in our Table 1 is formulation B, Tri-Sprintec has no letter due to missing data but is the norgestimate with three different dosages combined with 0.035 mg/day EE, and Sprintec is formulation D. Ideally more resources need to be directed toward research and development so that the safety of these and other established OCs can be better assured.

One alternative to OCs is ParaGard, a copper intrauterine contraceptive (IUC) that is nonhormonal. Risks are best understood when categorized by ‘typical’ use and ‘perfect’ use. Under ‘typical use’ the percentage of women experiencing an unintended pregnancy within the first year of use has been estimated at 0.8% for ParaGard versus 9% for OCs. Under perfect use, these risks are 0.6% and 0.3%, respectively [74]. Presently, IUCs are under-prescribed in the US, despite being a safe and effective birth control option [75].

Women’s hormonal profiles are greatly altered compared to those of the human evolutionary past prior to the demographic transition to low fertility and diminished breast feeding [3, 24]. The evolutionarily novel pattern of repeated menstrual cycling, and relatively high hormone levels, has been implicated in the increased prevalence of breast cancer [4]. We hope that our results will stimulate research on whether the additional novelty of replacing the endogenous ovarian cycle with exogenous hormones will lead to a further increase in breast cancer risk. Our study identified four OC formulations [containing levonorgestrel (A), norethindrone acetate (B) and drospirenone (F & G) in Table 1 and Fig. 4B] that more than quadruple progestin levels when compared to median endogenous exposure from progesterone for women who are not on the pill. We also found that another widely prescribed formulation containing norgestimate and 0.035 mg/day EE (formulation D in Table 1 and Fig. 4A) was 40% higher in EE exposure relative to median E2. We predict that these formulations will increase breast cancer risk and further research is needed to establish their safety.

## Supplementary Material

Supplementary Data
