# Supplementary material for: Oral contraceptives cause evolutionarily novel increases in hormone exposure: A risk factor for breast cancer
Source: Evol Med Public Health. 2017 Jun 5;2017(1):97–108. doi: 10.1093/emph/eox009 (PMC5494186; doi:10.1093/emph/eox009)
Supplement: Supplementary Data [file eox009_supp.zip › Supplementary Table 3.docx]

**Supplementary Table 3**. Review articles in the literature that reported relative binding affinities (%). The values in bold type are adjusted so that progesterone is the reference compound.

|  | (Burkman, Bell and Serfaty 2011) | (Mueck and Sitruk-Ware 2011) | (Kuhl 2005) | (Schindler *et al.* 2003) | (Kuhl 2001a) | (Kuhl 2001b) | (Rozenbaum 2002) | | (Africander, Verhoog and Hapgood 2011) | (Sitruk-Ware 2005) | (Sitruk-Ware 2008) | (Sitruk-Ware 2004) | (Winneker, Bitran and Zhang 2003) | (Elger *et al.* 2003) | (Ojasoo 1995) |
| --- | --- | --- | --- | --- | --- | --- | --- | --- | --- | --- | --- | --- | --- | --- | --- |
| **Authors cited** | Kuhl 2005 | Kuhl 2005 | Kumar 2000, Philibert 1999, Phillips 1990,Ojasoo 1995, Pollow et al. 1992 | Khul 2001a | Kuhl 2001b | Source not given^a^ | Kuhl 2001a | Philibert et al. 1999 and Kratten-  macher 2000 | Schindler et al. 2003^*^ and Philibert 1999** | Sitruk-Ware 2004 | Philibert et al. 1999 | Philibert et al. 1999 and Kratten-macher 2000 | Philibert et al. 1999 | Pollow et al. 1992 | Ray-naud & Bouton 1980 |
| **Progestins** |  |  |  |  |  |  |  |  |  |  |  |  |  |  |  |
| Levonorgestrel | 150  **300** | 150  **300** | 150  **300** | 150  **300** | 150  **300** | 150  **300** |  | 323 | 300*, 323** | 323 | 323 | 323 | 323 | 250  **833** |  |
| Norethindrone | 75  **150** | 75  **150** | 75  **150** | 75  **150** | 75  **150** | 75  **150** | 75  **150** | 134 |  | 134 | 134 | 134 | 134 |  | 265 |
| Desogestrel |  |  |  | 1  **2** |  | 1  **2** |  |  |  |  |  |  |  |  |  |
| Norgestimate | 15  **30** | 15  **30** | 15  **30** | 15  **30** | 15  **30** | 15  **30** |  |  |  |  |  |  |  |  |  |
| Drospirenone | 35  **70** | 35  **70** | 35  **70** | 35  **70** | 35  **70** | 40  **80** |  | 19 | 70*, 19** | 19 |  | 19 |  | 20  **67** |  |
| ^3^H-R5020 (promegestone) |  | 100  **200** | 100  **200** | 100  **200** | 100  **200** |  | 100  **200** |  |  |  |  |  |  |  |  |
| Progesterone | 50  **100** | 50  **100** | 50  **100** | 50  **100** | 50  **100** | 50  **100** | 50  **100** | 100 | 100 | 100 | 100 | 100 | 100 | 30  **100** | 100 |
| **Metabolites** |  |  |  |  |  |  |  |  |  |  |  |  |  |  |  |
| Levonorgestrel-3-oxime (17deacetyl.) |  |  |  | 10  **20** |  | 10  **20** |  |  |  |  |  |  |  |  |  |
| Levonorgestrel-17-acetate (3keto) |  |  |  | 135  **270** |  | 135  **270** |  |  |  |  |  |  |  |  |  |
| 3-ketodesogestrel (etonogestrel) | 150  **300** |  |  | 150  **300** | 150  **300** | 150  **300** |  |  |  |  |  |  |  | 180  **600** |  |

^a^(Kuhl 2001b) is a text book that gives RBA values for several progestins but does not provide the source of the primary data. Several review articles cite authors who in turn cite (Kuhl 2001b), however no source provides the primary data.
