# Supplementary material for: Oral contraceptives cause evolutionarily novel increases in hormone exposure: A risk factor for breast cancer
Source: Evol Med Public Health. 2017 Jun 5;2017(1):97–108. doi: 10.1093/emph/eox009 (PMC5494186; doi:10.1093/emph/eox009)
Supplement: Supplementary Data [file eox009_supp.zip › Supplementary Table 4.docx]

**Supplementary Table 4**. RBA of progestins to the androgen receptor (AR), estrogen receptor (ER), glucocorticoid receptor (GR), and mineralocorticoid receptor (MR) (Kuhl, 1990; Krattenmacher, 2000; Stanczyk, 2002; Schindler *et al.*, 2003; Kuhl and Kuhl, 2005; Africander *et al.*, 2011; Archer and Lasa, 2011; Wiegratz and Thaler, 2011)

| **Progestins (OC)** | AR | ER | GR | MR | Generation | Classification of progestin | Bioavailability^a^ | Ovulation inhibitory dose ^d^  (mg) |
| --- | --- | --- | --- | --- | --- | --- | --- | --- |
| Levonorgestrel (A) | 45 | <0.02 | 7.5 | 17 | 2nd | 19-Nortestosterone (13-ethylgonane) derivative | 100% | 0.06 |
| Norethindrone (B) | 15 | 0.15 | 1.4 | 2.7 | 1st | 19-Nortestosterone (estrane) derivative | 64%  50-77%  47-73% | 0.4 |
| Desogestrel (C) | 0 | 0 | 0 | 0 | 3rd | 19-Nortestosterone (13-ethylgonane) derivative | 76% | 0.06 |
| 3-ketodesogestrel (active metabolite in desogestrel) | 20 | 0 | 14 | 0 | — | — | — | — |
| Norgestimate (D,E) | 0 | 0 | 1 | 0 | 3rd | 19-Nortestosterone (13-ethylgonane) derivative | ^b^ | 0.2 |
| Drospirenone (F,G) | 65 | 0.5 | 3 | 500 | 4th | Spirolactone derivative | 76%  66% | 2 |
| Progesterone | 0 | 0.5 | 11 | 1000 |  | Natural | 5%^c^ | 300 |
|  |  |  |  |  |  |  |  |  |

Reference steroids are as listed: AR: metribolone = 100% affinity, ER: 17-β-estradiol = 100%, GR: dexamethasone = 100%, MR: aldosterone = 100%.

^a^ Bioavailability is defined as the extent to which the administered drug reaches the systemic circulation after undergoing hepatic first pass metabolism (Stanczyk 2002).

^b^ No accurate data are available on bioavailability because norgestimate is a pro-drug that is almost completely metabolized into its active metabolites levonorgestrel and levonorgestrel-3-oxime (norelgestromin).

^c^ Estimate of the bioavailability of micronized progesterone following oral dosing.

^d^ Minimal daily dose required to inhibit ovulation in all women tested (without added estrogen).
