# Supplementary material for: Oral contraceptives cause evolutionarily novel increases in hormone exposure: A risk factor for breast cancer
Source: Evol Med Public Health. 2017 Jun 5;2017(1):97–108. doi: 10.1093/emph/eox009 (PMC5494186; doi:10.1093/emph/eox009)
Supplement: Supplementary Data [file eox009_supp.zip › SI_refs.docx]

References for Supplementary Tables 2-4

Africander D, Verhoog N, Hapgood JP. Molecular mechanisms of steroid receptor-mediated actions by synthetic progestins used in HRT and contraception. *Steroids* 2011;**76**:636–52.

Archer DF, Lasa IL. Tailoring combination oral contraceptives to the individual woman. *J Womens Health (Larchmt)* 2011;**20**:879–91.

Bergink EW, Hamburger AD, De Jager E *et al.* Binding of a contraceptive progestogen Org 2969 and its metabolites to receptor proteins and human sex hormone binding globulin. *J Steroid Biochem* 1981;**14**:175–83.

Burkman R, Bell C, Serfaty D. The evolution of combined oral contraception: improving the risk-to-benefit ratio. *Contraception* 2011;**84**:19–34.

Elger W, Beier S, Pollow K *et al.* Conception and pharmacodynamic profile of drospirenone. *Steroids* 2003;**68**:891–905.

Fuhrmann U, Krattenmacher R, Slater EP *et al.* The novel progestin drospirenone and its natural counterpart progesterone: Biochemical profile and antiandrogenic potential. *Contraception* 1996;**54**:243–51.

Fuhrmann U, Slater EP, Fritzemeier K-H. Characterization of the novel progestin gestodene by receptor binding studies and transactivation assays. *Contraception* 1995;**51**:45–52.

Juchem N, Pollow K, Elger W *et al.* Receptor binding of norgestimate — A new orally active synthetic progestational compound. *Contraception* 1993;**47**:283–94.

Kasid A, Buckshee K, Hingorani V *et al.* Interaction of progestins with steroid receptors in human uterus. *Biochem J* 1978;**176**:531–9.

Killinger J, Hahn DW, Phillips A *et al.* The affinity of norgestimate for uterine progestogen receptors and its direct action on the uterus. *Contraception* 1985;**32**:311–9.

Krattenmacher R. Drospirenone: pharmacology and pharmacokinetics of a unique progestogen. *Contraception* 2000;**62**:29–38.

Kuhl H. Pharmacokinetics of oestrogens and progestogens. *Maturitas* 1990;**12**:171–97.

Kuhl H. [New gestagens--advantages and disadvantages]. *Ther Umsch* 2001a;**58**:527–33.

Kuhl H. Natürliche und synthetische Steroidhormone. In: Bender H, Diedrich K, Künzel W (eds.). *Endokrinologie Und Reproduktionsmedizin I*. Munchen: Urban & Fischer, 2001b, 68–101.

Kuhl H. Pharmacology of estrogens and progestogens: influence of different routes of administration. *Climacteric* 2005;**8 Suppl 1**:3–63.

Kumar N, Koide SS, Tsong Y *et al.* Nestorone (R) : a progestin with a unique pharmacological profile. *Steroids*. Vol 65. 2000, 629–36.

Mueck AO, Sitruk-Ware R. Nomegestrol acetate, a novel progestogen for oral contraception. *Steroids* 2011;**76**:531–9.

Ojasoo T. Multivariate Preclinical Evaluation of Progestins. *Menopause* 1995;**2**:97–107.

Philibert D, Bouchoux F, Degryse M *et al.* The pharmacological profile of a novel norpregnance progestin (trimegestone). *Gynecol Endocrinol* 1999;**13**:316–26.

Phillips A, Demarest K, Hahn DW *et al.* Progestational and androgenic receptor binding affinities and in vivo activities of norgestimate and other progestins. *Contraception* 1990;**41**:399–410.

Pollow K, Juchem M, Elger W *et al.* Dihydrospirorenone (ZK30595): a novel synthetic progestagen--characterization of binding to different receptor proteins. *Contraception* 1992;**46**:561–74.

Pollow K, Juchem M, Grill HJ *et al.* Gestodene: a novel synthetic progestin--characterization of binding to receptor and serum proteins. *Contraception* 1989;**40**:325–41.

Raynaud JP, Bouton MM. The design of estrogens and/or anti-estrogens on the basis of receptor binding. *Cytotoxic Estrogens in Hormone Receptive Tumors*. London, New York: Academic Press, 1980, 49–70.

Raynaud JP, Bouton MM, Moguilewsky M *et al.* Steroid hormone receptors and pharmacology. *J Steroid Biochem* 1980;**12**:143–57.

Rozenbaum H. *Les Progestatifs*. Paris: ESKA, 2002.

Schindler AE, Campagnoli C, Druckmann R *et al.* Classification and pharmacology of progestins. *Maturitas* 2003;**46**:7–16.

Schoonen WGEJ, Dijkema R, de Ries RJH *et al.* Human progesterone receptor A and B isoforms in CHO cells. II. Comparison of binding, transactivation and ed50 values of several synthetic (anti)progestagens in vitro in CHO and MCF-7 cells and in vivo in rabbits and rats. *J Steroid Biochem Mol Biol* 1998;**64**:157–70.

Sitruk-Ware R. *New Progestogens: A Review of Their Effects in Perimenopausal and Postmenopausal Women*., 2004:865–83.

Sitruk-Ware R. Pharmacology of different progestogens: the special case of drospirenone. *Climacteric* 2005;**8 Suppl 3**:4–12.

Sitruk-Ware R. Reprint of Pharmacological profile of progestins. *Maturitas*. Vol 61. 2008, 151–7.

Stanczyk FZ. Pharmacokinetics and potency of progestins used for hormone replacement therapy and contraception. *Rev Endocr Metab Disord* 2002;**3**:211–24.

Wiegratz I, Thaler CJ. Hormonale Kontrazeption – Was, wann, für wen? *Dtsch Ärzteblatt Int* 2011;**108**:495–506.

Winneker RC, Bitran D, Zhang Z. The preclinical biology of a new potent and selective progestin: Trimegestone. *Steroids*. Vol 68. 2003, 915–20.
