# Supplementary material for: Oral contraceptives cause evolutionarily novel increases in hormone exposure: A risk factor for breast cancer
Source: Evol Med Public Health. 2017 Jun 5;2017(1):97–108. doi: 10.1093/emph/eox009 (PMC5494186; doi:10.1093/emph/eox009)
Supplement: Supplementary Data [file eox009_supp.zip › Supplementary Table 1.docx]

| **Supplementary Table 1**. Characteristics of the populations that provided data on endogenous serum estradiol and progesterone used in Supplementary Figure 1. All studies included one menstrual cycle per woman and aligned the data with respect to the luteinizing hormone (LH) peak^1^. Ovulation identified based on mid-cycle LH peak; additional criteria used are indicated. | | | | | |
| --- | --- | --- | --- | --- | --- |
| **Study Author, Publication Year** | **# Women** | **Age (years)** | **Sample Characteristics** | **Location** | **Collection Frequency** |
| Groome *et al.* 1996 [37] | 6 | 25-39 | Healthy with normal menstrual cycles; no form of hormonal therapy within preceding 3 months. | Lothian, Scotland | daily |
| Stricker *et al.* 2006 [38] | 20 | 20-36 | Healthy with normal menstrual cycles and no use of oral contraceptives or other medications. Ovulation based on ultrasound. | Geneva, Switzerland | daily |
| Moghissi *et al.* 1972 [39] | 10 | 19-40 | Healthy women. | Detroit, USA | every 2 days before cycle day 10 and after day 20; daily during mid-cycle |
| Chiu *et al.* 1999 [40] | 20 | 23-40 | Healthy with a history of normal menstrual cycles; exclusion criteria included a history of endocrine, renal, or metabolic diseases, and the use of oral contraceptives during the 3 months prior to the study. | San Francisco, USA | 3 times per week |
| Muttukrishna *et al.* 1996 [41] | 5 | 22-30 | Regularly cycling. | United Kingdom | every 3 days for first 9 days, daily for next 7 days, then every 3 days |
| Welt *et al.* 1999 [42] | 23 | 20-34 | Normal prolactin and thyroid stimulating hormone levels, no medication, normal body mass index (BMI), no history of excessive exercise, no evidence of androgen excess, regular menstrual cycles with evidence of ovulation in the preceding cycle based on a serum progesterone level of more than 6 ng/mL. Ovulation based three of four of the following criteria: 1) LH peak; 2) midcycle FSH peak; 3) day of or after the midcycle E2 peak; 4) day that the progesterone doubled from baseline or reached 0.6 ng/mL. A subset of subjects (N=14) received ultrasounds to detect follicle development and confirm ovulation. | Boston, USA | daily |
| Zumoff *et al.* 1990 [43] | 8^2^ | 20-35 | Normal women with regular menstrual cycles; non-smokers. Exclusion criteria included obesity (>20% above ideal weight), a history of significant chronic illness, a history of ingestion of drugs known or suspected to alter hormone secretion or metabolism, a history of oral contraceptive use or pregnancy in the previous 6 months, or abnormality of liver, kidney, or thyroid function. | New York, USA | days 2, 4, 6, 8, 10, 11, 12, 13, 14, 15, 16, 17, I8, 20, 22, 24, and 26 of a menstrual cycle, counting the start of the menstrual flow as day 1 |
| Soules *et al.* 1998 [44] | 12 | 20-25 | Normal BMI; no medications; no endocrine abnormalities including hirsutism, galactorrhea, and thyroid disease; no history of infertility or recurrent spontaneous abortion; regular menstrual cycles, normal prolactin and testosterone levels. | Seattle, USA | daily |
| Dighe *et al.* 2005 [45] | 51 | 18-35 | Normal BMI; normal kidney and hepatic function; regular menstrual cycles, no oral contraceptives for at least 3 months prior to study, no regular medications; normal prolactin and testosterone levels. | Boston, USA | daily |
| Christin-Maitre *et al.* 1996 [46] | 11 | 20-36 | Healthy, taking no medications, luteal phases of at least 10 days and a midluteal progesterone level of at least 9 ng/mL. Ovulation based three of four of the following criteria: 1) LH peak; 2) midcycle FSH peak; 3) day of or after the midcycle E2 peak; 4) day that progesterone doubled from baseline or reached 0.6 ng/mL. | Boston, USA | daily |
| Muttukrishna *et al.* 1994 [47] | 5 | 27.6 ±3.6 | Regularly cycling. | Salford, United Kingdom | every 3 days for the first 10 days, every day for the next 7 days, then every 3 days until the onset of the next menses |
| Mishell *et al.* 1971 [48] | 10 | 20-28 | Regularly cycling. | Los Angeles, USA | daily |

^1^All studies measured estradiol, progesterone, lutenizing hormone, and follicle stimulating hormone except Zumoff *et al.* [43] who did not assay FSH, Dighe *et al.* [45] who did not assay progesterone, and Moghissi *et al.* [39] who did not measure serum estradiol. For Moghissi *et al.* [39] we used only the progesterone data.

^2^Data from 8 additional women identified as smokers in the study were not used.
