# Supplementary material for: Oral contraceptives cause evolutionarily novel increases in hormone exposure: A risk factor for breast cancer
Source: Evol Med Public Health. 2017 Jun 5;2017(1):97–108. doi: 10.1093/emph/eox009 (PMC5494186; doi:10.1093/emph/eox009)
Supplement: Supplementary Data [file eox009_supp.zip › Supplementary Table 2.docx]

**Supplementary Table 2**. *In vitro* binding assays from studies in the primary literature that measured the relative binding affinity (%) of progesterone and various synthetic progestins to the progesterone receptor. Most studies used the binding affinity of progesterone as the reference, but a few studies used Org 2050 or ^3^H-R5020. Reference compounds have RBAs of 100%. The values in bold type are adjusted so that progesterone is the reference. Bold boxes indicate that the source of the progesterone receptor was human uteri.

| **Study** | (Kasid *et al.* 1978) | (Bergink *et al.* 1981) | | (Raynaud and Bouton 1980) | (Raynaud *et al.* 1980) | (Killinger *et al.* 1985) | (Pollow *et al.* 1989) | (Pollow *et al.* 1992) | (Juchem *et al.* 1993) | (Phillips *et al.* 1990) | (Fuhrmann, Slater and Fritzemeier 1995) | (Fuhrmann *et al.* 1996) | (Schoonen *et al.* 1998) | (Philibert *et al.* 1999) | (Krattenmacher 2000) | (Kumar *et al.* 2000) |
| --- | --- | --- | --- | --- | --- | --- | --- | --- | --- | --- | --- | --- | --- | --- | --- | --- |
| Source of progesterone receptors | human uteri | human uteri | Dutch (belted) rabbits | New Zealand rabbit uteri | rabbit uteri | New Zealand rabbit uteri | human uteri | human uteri | human uteri | rabbit uteri | rabbit uteri | rabbit uteri | human uteri | human  utetri | human  uteri | Sprague- Dawley rats |
| **Progestins** |  |  |  |  |  |  |  |  |  |  |  |  |  |  |  |  |
| Levonorgestrel |  | 113*, 120**  **628***, **1500**** | 141*, 113**  **441***, **491**** |  |  | 95 | 120 **300** | 250  **833** | 250  **833** | 541 |  |  | 94  **671** | 323 |  | 100  **500** |
| Norethindrone | 98 ± 9 | 55*  **306** | 50*  **156** | 263 | 263 |  |  |  |  |  |  |  | 27  **193** | 134 |  |  |
| Desogestrel |  |  |  |  |  |  | 1  **2.5** |  | 1  **3.3** |  |  |  |  |  |  |  |
| Norgestimate |  |  |  |  |  | 9.8 |  |  | 0.8  **2.3** | 124 |  |  |  |  |  |  |
| Drospirenone |  |  |  |  |  |  |  | 20  **67** |  |  |  | 40 |  |  | 19 |  |
| Org 2050 (7-[^3^H]-Org 2050) |  | 100 | 100 |  |  |  |  |  |  |  |  |  | 100 |  |  |  |
| ^3^H-R5020 (prome-gestone) |  |  |  |  |  |  | 100  **250** | 100  **333** | 100  **333** |  |  |  |  |  |  |  |
| Progesterone | 100 | 18*, 8**  **100***, **100**** | 32*, 23**  **100***, **100**** | 100 | 100 | 100 | 40  **100** | 30  **100** | 30  **100** | 100 | 100 | 100 | 14  **100** | 100 | 100 | 20  **100** |
| **Metabolites** |  |  |  |  |  |  |  |  |  |  |  |  |  |  |  |  |
| Levonorgestrel-3-oxime (17deacetyl.) |  |  |  |  |  | 19 |  |  | 8  **27** | 94 |  |  |  |  |  |  |
| Levonorgestrel-17-acetate (3keto) |  |  |  |  |  | 84 |  |  | 110  **367** | 521 |  |  |  |  |  |  |
| 3-keto-desogestrel (etonogestrel) |  |  |  |  |  |  | 130 **325** | 180  **600** | 180  **600** | 849 | 125 |  | 228  **1629** |  |  | 220  **1100** |

* Incubated for 16 h at 4°C.

**Incubated for 1.5 h at 30°C.

^a^ Data were originally reported as IC50s in nM as follows: Progesterone = 5.7, Norgestimate = 58, Levonorgestrel = 6, Levonorgestrel-3-oxime = 30, Levonorgestrel-17-acetate = 6.8. We calculated the RBAs by dividing the IC50 of progesterone by the IC50s of each compound.
